# Guidance for reporting artificial intelligence technology evaluations for ultrasound scanning in regional anaesthesia (GRAITE‐USRA): an international multidisciplinary consensus reporting framework

**DOI:** 10.1111/anae.16733

**Published:** 2025-09-18

**Authors:** Xiaoxi Zhang, Jenny Ferry, David W. Hewson, Gary S. Collins, Matthew D. Wiles, Yi Zhao, Alexander P. L. Martindale, Michael Tomaschek, James S. Bowness, the GRAITE‐USRA Working Group, Adam J Dixon, Admir Hadzic, Alan Karthikesalingam, Alan Macfarlane, Alex Novak, Alex T. Sia, Alwin Chuan, Amit Pawa, Anthony E. Samir, Arun Nagdev, Ashokka Balakrishnan, Athmaja Thottungal, Benjamin Fox, Boyne Bellew, Carmit Shiran, Clara Lobo, Colin J. L. McCartney, Craig Loomis, Damon Kamming, Daniel Perry, David W. Hewson, Edward R. Mariano, Eleni Moka, Erik Smistad, Francois Retief, Gary Collins, Gwendolynn McCaulley, Hannah Richardson, Hugh Hemmings, Iyabo O. Muse, Jenny Ferry, Jennifer M. Weller, Juan Pablo Miranda Pantoja, Kariem El‐Boghdadly, Lopa Misra, Marc Van de Velde, Martin Benson, Matthew Davies, Matthew Wiles, Melissa L. Byrne, Mohamed Mostafa Mohamed, Nabil M. Elkassabany, Nat Haslam, Cailin Ng, Norman Kachel, Peter Merjavy, Rajnish K. Gupta, Rosemary M. G. Hogg, Rupert M. Pearse, Samuel Gluck, Sandra L. Kopp, Sebastian Layera, Simeon West, Simon Kos, Stefan Mörl, Steve Margetts, Suwimon Tangwiwat, Tim Meek, Toby Ashken, Tom E. F. Abbott, Utku Kaya, William Manson, Veena Graff

**Affiliations:** ^1^ Department of Anaesthesia, Chelsea and Westminster Hospital London UK; ^2^ Department of Anaesthesia Aneurin Bevan University Health Board Newport UK; ^3^ Unit of Injury, Recovery and Inflammation Science, School of Medicine University of Nottingham Nottingham UK; ^4^ Department of Anaesthesia and Critical Care Nottingham University Hospitals NHS Trust Nottingham UK; ^5^ Centre for Statistics in Medicine, Nuffield Department of Orthopaedics, Rheumatology and Musculoskeletal Sciences University of Oxford Oxford UK; ^6^ Department of Anaesthesia and Critical Care Sheffield Teaching Hospitals NHS Foundation Trust Sheffield UK; ^7^ Centre for Applied Health and Social Care Research Sheffield Hallam University Sheffield UK; ^8^ Newham University Hospital London UK; ^9^ King's College Hospitals NHS Foundation Trust London UK; ^10^ NHS Ayrshire and Arran Scotland; ^11^ Department of Anaesthesia University College London Hospitals NHS Foundation Trust London UK; ^12^ Department of Targeted Intervention University College London London UK

**Keywords:** evaluation, regional anaesthesia, reporting, ultrasound artificial intelligence

## Abstract

**Introduction:**

The application of artificial intelligence to enhance the clinical practice of ultrasound‐guided regional anaesthesia is of increasing interest to clinicians, researchers and industry. The lack of standardised reporting for studies in this field hinders the comparability, reproducibility and integration of findings. We aimed to develop a consensus‐based reporting guideline for research evaluating artificial intelligence applications for ultrasound scanning in regional anaesthesia.

**Methods:**

We followed methodology recommended by the EQUATOR Network for the development of reporting guidelines. Review of published literature and expert consultation generated a preliminary list of candidate reporting items. An international, multidisciplinary, modified Delphi process was then undertaken, involving experts from clinical practice, academia and industry. Two rounds of expert consultation were conducted, in which participants evaluated each item for inclusion in a final reporting guideline, followed by an online discussion.

**Results:**

A total of 67 experts participated in the first Delphi round, 63 in the second round and 25 in the roundtable consensus meeting. The GRAITE‐USRA reporting guideline comprises 40 items addressing key aspects of reporting in artificial intelligence research for ultrasound scanning in regional anaesthesia. Specific items include ultrasound acquisition protocols and operator expertise, which are not covered in existing artificial intelligence reporting guidelines.

**Discussion:**

The GRAITE‐USRA reporting guideline provides a minimum set of recommendations for artificial intelligence‐related research for ultrasound scanning in regional anaesthesia. Its adoption will promote consistent reporting standards, enhance transparency, improve study reproducibility and ultimately support the effective integration of evidence into clinical practice.

## Plain Language Summary

Doctors, scientists and companies are becoming more interested in using artificial intelligence (AI) to help with a type of pain‐blocking treatment called ultrasound‐guided regional anaesthesia. But it is hard to compare studies about this because they are not all written in the same way. Our goal was to create a clear list of rules for how to write these kinds of studies so that everyone can understand and trust the results. We followed expert advice on how to make good rules for research reporting. First, we looked at past research and talked to experts to come up with a list of things that should be included in these studies. Then, we asked a group of experts from different countries and jobs (like doctors, scientists and people from companies) what they thought. They shared their opinions in two rounds of surveys and one online group discussion. In the first round, 67 experts gave feedback. In the second round, 63 experts joined. Then, 25 experts came together for a group meeting. In the end, we created the GRAITE‐USRA guideline. It has 40 important points that researchers should include when writing about AI for ultrasound in regional anaesthesia. Some of these points, like how the ultrasound images are taken and how skilled the person using the machine is, are not covered in other guidelines. The GRAITE‐USRA guideline gives clear and simple rules to help people write better research about using AI in ultrasound for pain‐blocking treatments. If everyone follows these rules, it will be easier to understand, trust and use the results in real medical care.

## Introduction

Ultrasound‐guided regional anaesthesia uses real‐time visualisation of key anatomical structures and the inserted needle to facilitate precise deposition of local anaesthetic around specific nerves or into fascial planes, providing analgesia or anaesthesia. Ultrasound guidance enhances the safety and efficacy of peripheral nerve blocks compared with electrical nerve stimulation methods of nerve localisation [1]. It has also shown benefits in central neuraxial techniques [2].

Artificial intelligence (AI) is a field of computer science that enables computers to perform tasks traditionally associated with human intelligence [3]. Several recent studies present the case for AI applied to ultrasound scanning and the delivery of ultrasound‐guided regional anaesthesia [3, 4]. Applications of AI currently available include highlighting key image features; labelling anatomical structures; and identifying optimal needle insertion points [4]. However, there is substantial heterogeneity in the reporting of studies evaluating AI‐based devices in healthcare, including ultrasound‐guided regional anaesthesia [4, 5]. These inconsistences hinder robust evaluation; reproducibility of findings; comparison of similar technologies; comprehension among the intended audiences; and, ultimately, the safe and appropriate integration of AI technology into clinical practice [6]. Several AI‐specific reporting guidelines have been developed to improve transparency and consistency across different study designs and clinical specialties. These include Transparent Reporting of a Multivariable Prediction Model of Individual Prognosis or Diagnosis for Artificial Intelligence (TRIPOD+AI); Checklist for Artificial Intelligence in Medical Imaging (CLAIM); and Consolidated Standards of Reporting Trials for Artificial Intelligence (CONSORT‐AI) [7, 8, 9]. However, none of these guidelines address the key aspects associated with evaluating AI technologies in ultrasound‐guided regional anaesthesia specifically, such as descriptions of the ultrasound scanning protocol; strategies for selecting anatomical features; and the expertise of those establishing the reference standard.

The aim of this study was to develop a reporting framework for studies evaluating AI technologies applied to ultrasound scanning in regional anaesthesia. Using a modified Delphi process, we sought to achieve consensus among an international, multidisciplinary group of stakeholders to establish a standardised framework for reporting studies in this rapidly evolving field.

## Methods

We established a steering group to oversee the reporting guideline development process. This consisted of members with expertise in ultrasound‐guided regional anaesthesia; Delphi consensus methodology; AI; reporting guideline development; and academic publication (XZ, JF, DH, GC, MW and JB).

A search of the EQUATOR Network database confirmed that no similar guidelines were already under development [10]. Following this, the project was registered as a reporting guideline initiative with the EQUATOR Network [11]. The guideline was developed subsequently in accordance with the methodological framework of the EQUATOR Network [10]. Definitions of key terms are provided in online Supporting Information Appendix S2. Ethical approval was not required, as determined by the University of Oxford and the UK Health Research Authority decision tool (online Supporting Information Appendices S3 and S4).

A comprehensive longlist of candidate items was developed through scrutiny of published literature and iterative consultation within the steering group. A recent systematic review of reporting guidelines for AI‐based tools in healthcare was used to identify existing guidelines [12]. In addition, we searched the EQUATOR Network on 30 May 2024 using the terms ‘artificial intelligence’, ‘machine learning’ and ‘deep learning’ to capture further relevant AI reporting guidelines that were not included in the systematic review. Guidelines still under development were not included for consideration.

A total of 33 reporting guidelines were identified [7, 8, 9, 13, 14, 15, 16, 17, 18, 19, 20, 21, 22, 23, 24, 25, 26, 27, 28, 29, 30, 31, 32, 33, 34, 35, 36, 37, 38, 39, 40, 41, 42]. Of these, 11 were subspecialty‐specific and not applicable to regional anaesthesia and were therefore not included [32, 33, 34, 35, 36, 37, 38, 39, 40, 41, 42]. From the remaining 22 relevant guidelines [7, 8, 9, 13, 14, 15, 16, 17, 18, 19, 20, 21, 22, 23, 24, 25, 26, 27, 28, 29, 30, 31], reporting recommendations were extracted independently and categorised by two investigators (YZ, APLM), with arbitration by a third (MT). A fourth investigator (JF) oversaw this process to ensure consistency, appropriate grouping and completeness. The resulting items underwent three rounds of iterative review by the steering group. Items were retained if they related to the evaluation of AI applications and were applicable to ultrasound‐guided regional anaesthesia. Items were not included if they were duplicative; highly subspecialty‐specific without relevance to ultrasound‐guided regional anaesthesia; or out of scope (e.g. addressing only model development, model architecture or model training rather than evaluation). As no previous reporting guideline exists for ultrasound‐guided regional anaesthesia, specific considerations and gaps were identified and incorporated as needed. Based on this process, a longlist of 43 candidate items tailored for AI applications in ultrasound imaging in regional anaesthesia was generated. This longlist (online Supporting Information Appendix S5) was structured according to the manuscript sections in which the items would appear and formed the basis of the modified Delphi process described below.

The steering group implemented a modified Delphi process [43], adopting a methodology based on recent Delphi studies in regional anaesthesia [44, 45, 46, 47, 48]. This work is reported in accordance with established guidelines for the reporting of Delphi studies [49].

International experts were invited to form the GRAITE‐USRA Working Group and participate in the online Delphi survey to vote on candidate items, refine item wording and propose additional items. Experts were identified and contacted by the steering group through a structured approach to ensure representation from all relevant stakeholder groups and promote diversity across disciplines, geographical regions and professional backgrounds. Selection criteria included authorship of relevant publications, recognised expertise in the field, recommendation within professional networks and nominations by endorsing societies. The Delphi panel included stakeholders from the following domains: practicing anaesthetists with subspecialty expertise in ultrasound‐guided regional anaesthesia; academic publishing and science dissemination, including journal editors and researchers in clinical medicine, medical imaging, life sciences, AI, statistics, computing science and engineering; and industry, comprising representatives from commercial entities active in the fields of healthcare AI and/or developing ultrasound‐guided regional anaesthesia devices with AI capability.

While commercial representation was deemed important for balanced stakeholder engagement, all experts were asked to declare potential conflicts of interest. These were documented and reviewed carefully by the steering group. To ensure transparency and diversity of perspectives, demographic details including sex, country of practice and professional role were recorded. The expertise and experience of each Delphi participant were verified by the steering group before formal invitation. Experts who agreed to participate were provided with a study summary detailing the aims and scope of the study, contact information for the study team, instructions on how to participate and a survey link. No financial incentives were offered for participation in the Delphi process. Further details are available in online Supporting Information Appendix S1.

The Delphi rounds were conducted online, in English, using Google Forms (Google, Mountain View, CA, USA), with each participant completing the surveys independently. To maintain confidentiality, all responses were anonymised before data analysis. A target response rate of 70% was set for each online survey voting round. Before the first round, a pilot survey was conducted with six individuals, including anaesthetists, academics and industry representatives, to assess usability and content. These individuals were not part of the subsequent Delphi panel, and their responses were not included in the final analysis.

Delphi participants were asked to evaluate candidate items in each round using the question stem: “*Should [candidate item] be included in the [section] of the reporting guideline?*” Candidate items were categorised under relevant manuscript sections such as ‘Title’; ‘Abstract’; ‘Methods’; ‘Results’; and ‘Discussion’. Panel members were asked to rate each candidate item using a four‐point Likert scale: 1 – definitely include; 2 – probably include; 3 – probably exclude; and 4 – definitely exclude. Items receiving ≥ 75% of responses as ‘definitely include’ were taken forward for inclusion into the final reporting guideline and not included in further rounds. Items for which > 50% of responses were ‘probably exclude’ or ‘definitely exclude’ were considered rejected by expert consensus and removed from subsequent rounds. The remaining items were retained for further Delphi rounds. At each stage, Delphi panel members could provide free text comments to offer additional insights or suggest new items. After each round, the steering group reviewed both the quantitative results and qualitative feedback, modifying candidate items or introducing new ones where necessary to reflect expert recommendations. Anonymised results, including free text responses and steering group decisions, were shared with all Delphi panel members to ensure transparency and encourage further engagement. Depending on the level of expert endorsement, up to three Delphi rounds were planned, followed by a final roundtable discussion to ratify the results with the expert panel, thus concluding the process. The final guideline, along with the study manuscript, was then circulated to all contributors for feedback and approval.

## Results

Between June 2024 and August 2024, 70 experts were identified and accepted the invite to participate in the modified Delphi process: 23 anaesthetists; 24 from academia and scientific publishing; and 23 representatives from the commercial sector. Characteristics of the Delphi participants are shown in Table 1.

**Table 1:** Characteristics of the Delphi experts. Values are number (proportion).

Characteristic	Round 1 (n = 67) 14 October to 3 November 2024	Round 2 (n = 63) 18 November to 8 December 2024	Roundtable (n = 25) 7 February 2025
**Sex**			
Female	20 (30%)	16 (25%)	7 (28%)
Male	47 (70%)	47 (75%)	18 (72%)
**Geographic location of current primary residence and work**			
Africa	3 (5%)	2 (3%)	2 (8%)
Asia	6 (9%)	6 (10%)	2 (8%)
Europe	33 (49%)	31 (49%)	18 (72%)
North America	20 (30%)	19 (30%)	2 (8%)
Oceania	4 (6%)	4 (6%)	0
South America	1 (2%)	1 (2%)	1 (4%)
**Domain of expertise**			
Anaesthetist with specialism in regional anaesthesia	23 (34%)	22 (35%)	12 (48%)
Academic publishing and science dissemination	24 (36%)	22 (35%)	10 (40%)
Industry	20 (30%)	19 (30%)	3 (12%)

The first Delphi round was completed by 67 experts (96% response rate). A total of 17 candidate items met the prespecified consensus threshold and were accepted into the final reporting guideline. Based on expert free text comments, the wording of three candidate items was revised for clarity, and three new candidate items were introduced to address additional considerations. No candidate items were rejected, leaving 29 items for evaluation in the second Delphi round.

The second Delphi round was completed by 63 experts (94% response rate). An additional six candidate items reached the consensus threshold and were accepted into the final reporting guideline, leaving 23 candidate items on the longlist for further consideration. No candidate items were rejected in this round. Based on expert free text comments, 21 items were refined to improve clarity and alignment with expert feedback.

Given the minimal variation in item endorsement between the first and second rounds, the steering group deemed it unlikely that a third Delphi round would yield significantly different rating patterns. Therefore, following the second round, the steering group opted to proceed directly to a roundtable discussion. The steering group also decided to include all 23 remaining candidate items in the draft guideline for further discussion with the Delphi experts, as even the lowest rated item had received a combined approval rating of 71% (‘definitely include’ or ‘probably include’).

**Figure 1:**
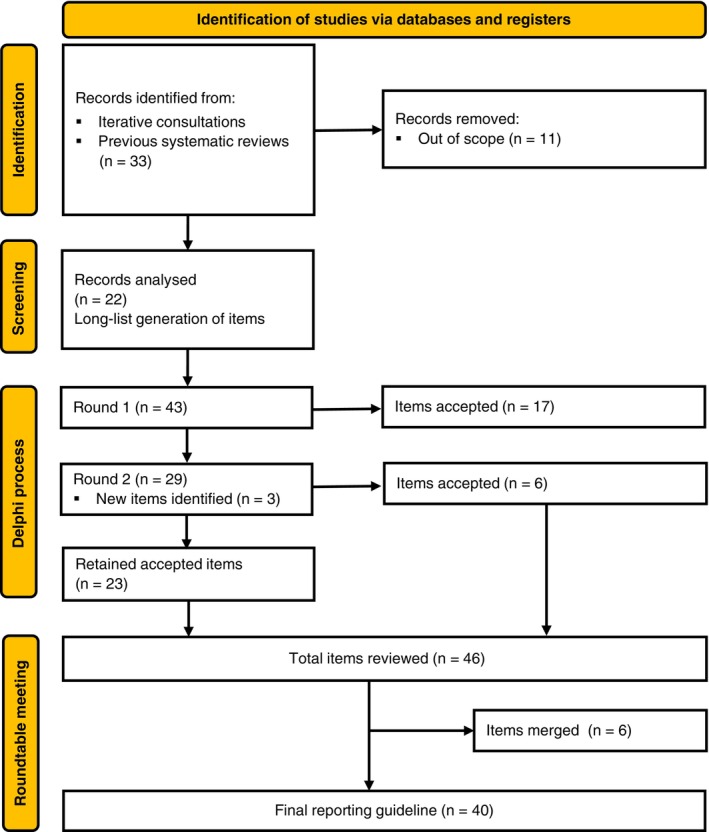
Flow chart showing data progress over study.

All 63 experts who participated in both Delphi rounds were invited to a roundtable discussion, held on 7 February 2025, via Microsoft Teams, with 25 experts in attendance. Those experts unable to attend were invited to submit written feedback on the provisional final checklist. At the roundtable discussion, 46 items were reviewed, and attendees were encouraged to comment on every checklist item; areas of uncertainty were also addressed. During these discussions, four items were re‐worded to improve clarity and six items were condensed and merged with others, resulting in a final checklist of 40 reporting items (Fig. 1). The steering group then conducted a review of the final checklist, ensuring language consistency and harmonisation across the guideline.

The GRAITE‐USRA comprises a 40‐item checklist designed specifically for reporting AI applications in ultrasound imaging for regional anaesthesia (Table 2). These are intended for reporting evaluations of AI technologies but do not prescribe methods for the design or development of these technologies.

**Table 2:** Guidance for reporting artificial intelligence technology evaluations in ultrasound scanning for regional anaesthesia (GRAITE‐USRA) checklist.

Section/topic	Item number	Checklist item	Page*
**TITLE**			
Title	1	State the study design, specify that the study involves ultrasound scanning in the context of regional anaesthesia and indicate that the intervention incorporates AI or machine learning.	
**ABSTRACT**			
Abstract	2	Summary of relevant background, aims, methods, study type, main results and conclusions.	
**INTRODUCTION**			
Background	3	Describe the current practice, standard of care or approach that represents the state of the art, and provide rationale for the study.	
Objectives	4	State the study objectives or hypotheses.	
**METHODS**			
Ethical approval	5	Provide details of ethical approval, informed participant consent (or assent) and compliance with regulatory requirements (e.g. trial registration, data security).	
Study design	6	Describe the overall study design (e.g. randomised controlled trial, cohort study, prospective/retrospective).	
AI intervention	7	Provide background on the AI intervention, including AI techniques used (e.g. deep learning), model outputs (e.g. classification, prediction) and version.	
Evaluation stage	8	Specify the evaluation stage (e.g. internal validation/testing or external validation) and provide details of the evaluation process.	
Participants	9	Describe the study setting, including number and location of centres.	
	10	Describe eligibility criteria separately for scan operators and scanned participants.	
Human factors	11	Describe any human factors considerations in the study design (e.g. providing training for scan operators, prior experience with AI intervention)	
Target population	12	Describe the intended target population (e.g. users and recipients of the AI intervention).	
Data	13	Describe the protocol for acquiring the ultrasound data, including details of the ultrasound machine manufacturer(s) and transducer(s) used.	
	14	Describe or justify the strategy to select features used for evaluation (e.g. block region, anatomical structures).	
Outcome	15	Define all primary and secondary outcome measures, including how and when they were assessed.	
Reference standard	16	Define and justify the reference standard (ground truth) used to evaluate the AI intervention (e.g. the number and expertise of the sources or reviewers involved).	
Analysis	17	Specify all measures used to evaluate the AI model performance and explain why these specific measures were chosen.	
	18	Describe the methods for statistical analyses used to evaluate both primary and secondary outcomes.	
Sample size	19	Describe how the sample size was determined, including details of any calculations and the clinical and statistical assumptions used.	
Adverse events	20	Report how any adverse events were defined.	
Stakeholder involvement	21	Provide details on any patient, public or stakeholder involvement in the design, execution, reporting, interpretation or sharing of the study results.	
**RESULTS**			
Data	22	Report dates of data collection.	
Participants	23	Describe the flow of participants through the study (e.g. enrolment, allocation to intervention, follow‐up, analysis), using a diagram if helpful for clarity.	
	24	Report the characteristics on the scan operators (e.g. level of training in regional anaesthesia) and scanned participants (e.g. age, sex, BMI, ethnicity).	
Outcomes	25	Report results in a manner consistent with primary and secondary outcomes (including results of prespecified statistical analyses).	
Missing data	26	Report missing data (e.g. lost data, non‐adherence to protocol).	
Adverse events	27	Report frequency and severity of any adverse events.	
**DISCUSSION**			
Interpretation	28	Describe the key findings related to the AI intervention in the target population (e.g. patients, clinicians and other potential stakeholders).	
	29	Provide a general and balanced interpretation of results with reference to study objectives.	
	30	Place findings in context of previous studies/current landscape (including benchmarking against other available data).	
Strengths and limitations	31	Discuss the strengths and limitations of the study and the AI intervention, including potential facilitators and barriers to implementation.	
Future work	32	Discuss implications and potential for future work.	
Conclusion	33	Provide a conclusion of the study, summarising the main findings and their potential impact.	
**OPEN SCIENCE**			
Adherence to standards	34	Adhere to community‐defined standards (e.g. Delphi consensus recommendations on nomenclature and anatomical structures).	
Conflicts of interest	35	Declare any conflicts of interest and financial disclosures for all authors.	
Funding	36	Specify the source of funding and the role of the funders for the current study.	
Protocol	37	State where the study protocol can be accessed, or specify no protocol was prepared.	
	38	Report any substantial amendments to the study protocol made after study commencement.	
Data	39	Provide details of the availability of the data collected during the study. If they are not accessible, provide reasons why.	
Code	40	Provide details of the availability of the AI intervention and analytical code. If it is not accessible, provide reasons why (e.g. intellectual property protection).	

* Record the page number the information is reported and write N/A if the item is not applicable.

We recommend applying the GRAITE‐USRA checklist during study planning and at an early stage in manuscript preparation to ensure essential details are considered and reported adequately. To facilitate implementation, an expanded checklist with rationale and guidance for each item is provided in online Supporting Information Appendix S6. The checklist establishes a minimum standard for clear, consistent and comprehensive reporting of studies. Authors are encouraged to provide additional relevant information and, depending on specific journal requirements, may report some checklist items in supplementary material or by referring to the study protocol, provided it is publicly accessible.

The steering group and Delphi experts organised the items in a logical sequence reflecting the natural progression of reporting in a research manuscript. However, item order may be adjusted to comply with specific journal formatting requirements and we therefore do not mandate the precise placement of each recommendation within a publication. To aid the editorial and peer review process, we recommend authors submit a completed checklist, clearly indicating the page number on which each reporting item can be found. For items deemed not applicable to a particular study (e.g. where information is irrelevant), authors should enter ‘N/A’ in the checklist and acknowledge this explicitly in the manuscript. Where information related to specific reporting items is protected by intellectual property restrictions, the steering group consider it acceptable to state this in the completed checklist; this guideline is intended to inform reporting and not to discourage potential commercial engagement in the academic process. Updates relating to GRAITE‐USRA will be disseminated through the EQUATOR Network. To enhance accessibility and inclusivity, enquiries to translate the checklist into other languages are welcome. Those interested should contact the corresponding author and follow a structured translation process involving the authors of the original publication, whose approval should be obtained.

## Discussion

Artificial intelligence will play an increasingly significant role in anaesthesia, critical care, peri‐operative medicine and pain management [50]. An international Delphi study on research priorities in regional anaesthesia identified advancement of the specialty through novel technology as a top 10 priority [47]. However, a scoping review examining studies on AI‐based tools for ultrasound scanning in regional anaesthesia highlighted the lack of comprehensive reporting standards tailored specifically to the evaluation of AI applications in this field [4]. Our has work addressed this gap. By standardising reporting practices, this guideline aims to enable clinicians to critically appraise AI technologies, compare and synthesise trial data and guide evidence‐based adoption into clinical practice. Additionally, clearer reporting may inform hospital equipment procurement decisions by providing a transparent assessment of AI‐based ultrasound‐guided regional anaesthesia technologies. We anticipate that this guideline will also be valuable for industry stakeholders, particularly commercial entities submitting reports for regulatory approval or presenting AI technologies to clinical audiences. Standardised reporting will facilitate more rigorous evaluation by regulatory bodies and improve the clarity of information provided to end users.

The GRAITE‐USRA checklist is designed specifically for AI applications in ultrasound imaging for regional anaesthesia, addressing both common and unique challenges in this field. It is intended to complement, rather than replace, existing reporting guidelines and should be regarded as a minimum standard for ensuring clear, consistent and comprehensive documentation of a study. The steering group agreed on the importance of retaining broader reporting items that may appear in other checklists, as the GRAITE‐USRA guideline is intended to function as a stand‐alone document, rather than an additional set of requirements used alongside a more general reporting framework. The GRAITE‐USRA checklist therefore includes several reporting recommendations (e.g. Item 4: “*State the study objectives or hypotheses*”, online Supporting Information Appendix S6) which can be considered generic and feature in many comparable reporting guidelines, including CONSORT‐AI. Importantly for standardisation of regional anaesthesia AI‐related research, GRAITE‐USRA contains several entirely unique reporting items which can be considered bespoke to regional anaesthesia research (such as Item 14: “*Describe or justify the strategy to select features used for evaluation (e.g. block region, anatomical structures)*”, online Supporting Information Appendix S6) and contextualises many of the generic reporting items with accompanying explanatory text to inform researchers of the correct implementation of such items into AI‐related regional anaesthesia research.

A notable strength of this study was the high response rates achieved from all participant groups, reflecting strong engagement across diverse areas of expertise. Response rates were consistently high, with anaesthetists achieving 95% (22/23), academics 92% (22/24) and industry representatives 83% (19/23), representing commitment across all groups to the development of the guideline. This robust stakeholder engagement has contributed to a comprehensive and representative set of recommendations, strengthening the validity and applicability of the final guideline.

When considering endorsement of this project, an issue noted by one specialist society was the potential to introduce conflict of interests through industry participation. Healthcare AI will unavoidably and necessarily involve industry participation at all stages due to the financial and technical resources in this sector, in addition to the commercial expertise necessary to realise ideas as medical devices ready to use. Indeed, there are concerns that this field will become industry‐led and fail to fully meet the needs of patients and healthcare professionals [50]. As such, the steering group believe commercial representation was essential to ensure appropriate representation of stakeholder categories. However, it is imperative for the clinical community to take a leadership role in shaping the healthcare AI landscape. Without active engagement, healthcare professionals risk becoming passive recipients of technology that may not fully align with clinical workflows or patient care priorities. Moving forward, efforts are needed on both sides to strengthen dialogue, ensuring clinical co‐leadership so that industry activities address real‐world clinical challenges [50].

The lack of patient and public involvement is acknowledged as a limitation of this study. As this was an unfunded project, there were no allocated resources available to facilitate patient and public involvement; however, we recognise the value of patient, carer and multidisciplinary team engagement [51]. Future guideline initiatives may benefit from incorporating broader stakeholder perspectives to enhance relevance, acceptability and applicability. Another limitation is the under‐representation of experts from low‐ and middle‐income countries, with the majority of Delphi experts based in high‐income regions such as Europe and North America. Although the project received endorsement from professional societies in Africa and Latin America, the number of contributing experts from these regions was relatively low. This may reflect wider global disparities in access to technology, AI infrastructure or established expertise in regional anaesthesia and research. Greater inclusion of low‐ and middle‐income country perspectives in future updates of the guideline will be important to ensure global relevance and equity.

A further consideration is that none of the candidate items were rejected in the Delphi process. This likely reflects the rigorous approach taken to generating the initial longlist, which was based on existing reporting guidelines and refined through iterative consultations with the steering group. This study aimed to harmonise existing reporting frameworks, adapting them for the specific context of AI applications in ultrasound scanning for regional anaesthesia.

Finally, we anticipate that this guideline will require periodic updates to remain aligned with advancements in AI technology. The rapid pace of AI development means that new methodologies, regulatory considerations and clinical applications will emerge, necessitating ongoing refinement of reporting standards. Future guideline revisions should be responsive to technological and clinical shifts, ensuring that reporting standards continue to facilitate transparent, reproducible and clinically relevant research.

While this guideline focuses specifically on AI applications in ultrasound scanning for regional anaesthesia, AI is being integrated increasingly into varied aspects of anaesthesia, peri‐operative medicine and pain management. Similar reporting frameworks should be developed for other AI applications in healthcare, ensuring that clinicians remain at the forefront of the evaluation and implementation of novel AI technologies. Beyond standardising reporting, future consensus‐driven initiatives could also help identify areas of clinical practice that would benefit from AI development, guiding research and innovation towards high‐impact clinical applications. Establishing clinically driven priorities in AI development is essential to ensure that emerging technologies align with real‐world patient care needs, enhance safety and efficiency and integrate seamlessly into existing clinical workflows.

Ultimately, fostering stronger collaboration between clinicians, researchers, regulators and industry partners will be key to shaping the future of AI in healthcare. While industry will inevitably undertake much of healthcare AI development, clinicians must take an active leadership role, ensuring that AI technologies are designed and evaluated in a way that is meaningful to clinicians, meet regulatory standards, address real‐world clinical challenges and enhance patient care.

In summary, GRAITE‐USRA provides a set of minimum recommendations for reporting evaluations of AI technology for ultrasound scanning in regional anaesthesia. It is hoped that this will be adopted and utilised in future academic studies pertaining to this field, potentially used in commercial settings, for regulatory review and expanded to other relevant domains as the field of healthcare AI evolves.

## Supporting information


**Plain Language Summary**.

**Appendix S1.** GRAITE‐USRA Working Group.

**Appendix S2.** Glossary of terms.
**Appendix S3.** Oxford University ethics waiver.
**Appendix S4.** HRA Decision Tool.
**Appendix S5.** GRAITE‐USRA longlist.
**Appendix S6.** GRAITE‐USRA expanded checklist.
