# Supplementary material for: Guidance for reporting artificial intelligence technology evaluations for ultrasound scanning in regional anaesthesia (GRAITE‐USRA): an international multidisciplinary consensus reporting framework
Source: Anaesthesia. 2025 Sep 18;80(12):1528–39. doi: 10.1111/anae.16733 (PMC12614416; doi:10.1111/anae.16733)
Supplement: Supplementary file 1 — Plain Language Summary. [file ANAE-80-1528-s003.docx]

**Plain Language Summary**
Doctors, scientists, and companies are becoming more interested in using artificial intelligence (AI) to help with a type of pain-blocking treatment called ultrasound-guided regional anaesthesia. But it’s hard to compare studies about this because they aren’t all written in the same way. Our goal was to create a clear list of rules for how to write these kinds of studies so that everyone can understand and trust the results. We followed expert advice on how to make good rules for research reporting. First, we looked at past research and talked to experts to come up with a list of things that should be included in these studies. Then, we asked a group of experts from different countries and jobs (like doctors, scientists, and people from companies) what they thought. They shared their opinions in two rounds of surveys and one online group discussion. In the first round, 67 experts gave feedback. In the second round, 63 experts joined. Then, 25 experts came together for a group meeting. In the end, we created the GRAITE-USRA guideline. It has 40 important points that researchers should include when writing about AI for ultrasound in regional anaesthesia. Some of these points, like how the ultrasound images are taken and how skilled the person using the machine is, are not covered in other guidelines. The GRAITE-USRA guideline gives clear and simple rules to help people write better research about using AI in ultrasound for pain-blocking treatments. If everyone follows these rules, it will be easier to understand, trust, and use the results in real medical care.
