# Supplementary material for: Guidance for reporting artificial intelligence technology evaluations for ultrasound scanning in regional anaesthesia (GRAITE‐USRA): an international multidisciplinary consensus reporting framework
Source: Anaesthesia. 2025 Sep 18;80(12):1528–39. doi: 10.1111/anae.16733 (PMC12614416; doi:10.1111/anae.16733)
Supplement: Supplementary file 2 — Appendix S1. GRAITE‐USRA Working Group. [file ANAE-80-1528-s002.pdf]

Appendix 1 GRAITE-USRA Working Group

Name	Affiliations	COI
Adam J Dixon	Executive Vice President of Engineering, Rivanna Medical, Inc.	Owner of Equity in Rivanna Medical, Inc; Inventor on several patents relevant to this topic
Admir Hadzic	Anesthesiologist Ziekenhuis Oost-Limburg, Genk, Belgium (MD, PhD) 1Department of Anesthesiology, Ziekenhuis Oost-Limburg, Genk, Belgium 2 Department of Anesthesiology & Algology, Faculty of Medicine, KU Leuven, Leuven, Belgium	AH has consulted, advised and/or performed industry-sponsored research for Philipps, GE, Sonosite, Konica Minolta, Codman & Shrutleff (Johnson and Johnson), Cadence, Insitu Biologics, Heron Therapeutics, Pacira, Baxter and BBraun Medical. AH receives royalty income from BBraun Medical. He owns and directs the New York School of Regional Anesthesia.
Alan Karthikesalingam	Research Scientist, Google	
Alan Macfarlane	Consultant Anaesthetist, Glasgow Royal Infirmary; Honorary Professor, University of Glasgow	Honorarium from GE Healthcare; Consultancy fees from Intelligent Ultrasound
Alex Novak	1) Consultant in Emergency Medicine and Ambulatory Care, Oxford University Hospitals NHS Foundation Trust, 2) Director of Emergency Medicine Research Oxford, (EMROx), 3) Associate Professor, Royal College of Emergency Medicine	Consultation for GE Healthcare Ltd (2021)
Alex T Sia	CEO and Professor, KK Women's and Children's Hospital, Singapore	CoFounder, HiCura Pte Ltd
Alwin Chuan	Associate Professor, South West Sydney Clinical Campus., Faculty of Medicine & Health, UNSW Sydney, Australia	Speaking and consulting honoraria from GE Healthcare Australia
Amit Pawa	Consultant Anaesthetist, Guy's & St Thomas' NHS Foundation Trust & Cleveland Clinic London<br><br>Clinical Professor of Anesthesiology, Cleveland Clinic Lerner College of	Honoraria from GE Healthcare for Teaching<br>Consultancy for Pacira Pharmaceuticals

	Medicine of Case Western Reserve University	
Anthony E. Samir	(1) Associate Chair, Medical Imaging Sciences, Massachusetts General Hospital; (2) Associate Professor of Medicine, Harvard Medical School	A.E.S. has served as a compensated consultant for Astra Zeneca, Bracco Diagnostics, Bristol Myers Squibb, General Electric, Gerson Lehman Group, Guidepoint Global Advisors, Supersonic Imagine, Novartis, Pfizer, Philips, Parexel Informatics, and WorldCare Clinical. He holds stock options in Rhino Healthtech Inc and Resolve Stroke SAS. He holds advisory board or committee memberships for General Electric and the Foundation for the National Institutes of Health. He is a member of the Board of Governors of the American Institute for Ultrasound in Medicine. His institution has received research grant support and equipment for projects that he has led from the Analogic Corporation, Canon, Echosens, General Electric, Hitachi, Philips, Siemens, Supersonic Imagine/Hologic, Toshiba Medical Systems, the US Department of Defense, Fujifilm Healthcare, the Foundation for the National Institutes of Health, Partners Healthcare, Toshiba Medical Systems, and Siemens Medical Systems. He holds equity in Avira Inc., Autonomus Medical Technologies, Inc., Evidence Based Psychology LLC, Klea LLC, Katharos Laboratories LLC, Ochre Bio, Inc., Quantix Bio LLC, and Sonoluminous LLC. He receives royalties from Elsevier Inc. and Katharos Laboratories LLC.
Arun Nagdev	Director, Emergency Ultrasound, Alameda Health System; Associate Clinical Professor, Department of Emergency Medicine, University of California, San Francisco	VP of Clinical, Exo Inc.
Ashokka Balakrishnan	Senior Consultant Anaesthesiologist, Clinical A/Prof; National University Hospital Singapore	
Athmaja Thottungal	Consultant, Anaesthesia and Pain management, Kent and Canterbury Hospital, East Kent University Hospitals NHS Trust	
Benjamin Fox	Consultant anaesthetist and Divisional Director Queen Elizabeth Hospital Kings	Hospitality from Edwards Life Science and honorarium from Medovate and Sintetica

	Lynn, Board Member Association of Anaesthetists	
Boyne Bellew	Consultant Anaesthetist, Royal National Orthopaedic Hospital, Stanmore<br>Honorary Clinical Senior Lecturer, Imperial College London	Honoraria from Intelligent Ultrasound Limited
Carmit Shiran	Director Clinical Insights & Development, PoC & HH GE Healthcare	Director Clinical Insights & Development, PoC & HH GE Healthcare
Clara Lobo	Anesthesiology Consultant	No conflicts
Colin J.L. McCartney	Chief of Anesthesia, Sunnybrook Health Sciences Centre, Professor of Anesthesiology and Pain Medicine, University of Toronto, ON, Canada	No conflicts
Craig Loomis	Senior Director of Product Management, RIVANNA	
Damon Kamming	Consultant Anaesthetist University College London Hospitals NHS Foundation Trust	
Daniel Perry	NIHR Research Professor, University of Liverpool	
David W Hewson	Associate Professor, University of Nottingham<br>Honorary Consultant Anaesthetist, Nottingham University Hospitals NHS Trust	
Edward R Mariano	Professor and Vice Chair, Department of Anesthesiology, Perioperative and Pain Medicine, Stanford University School of Medicine, Stanford, CA, USA; Chief, Anesthesiology, Perioperative and Pain Medicine Service, Veterans Affairs Palo Alto Health Care System, Palo Alto, CA, USA	Editor of Anaesthesia

Eleni Moka	Consultant Anaesthesiologist, Creta Interclinic Hospital, Hellenic Healthcare Group (HHG), Heraklion, Crete, Greece<br>ESRA President	President of the European Society of Regional Anaesthesia and Pain Therapy (ESRA)<br>Lecture fees from MENARINI Hellas
Erik Smistad	Research Scientist at SINTEF Health and at the Norwegian University of Science and Technology	GE HealthCare is an industrial partner in a research center hosted by the Norwegian University of Science and Technology.
Francois Retief	Head Clinical Unit. Department of Anaesthesiology and Critical Care, Faculty of Medicine and Health Sciences, Stellenbosch University	
Gary Collins	Professor of Medical Statistics, UK EQUATOR Centre/Centre for Statistics in Medicine, University of Oxford	National Institute for Health and Care Research (NIHR) Senior Investigator. The views expressed in this article are those of the author(s) and not necessarily those of the NIHR, or the Department of Health and Social Care.
Gwendolynn McCaulley	Access and OB Clinical Product Manager Philips	
Hannah Richardson	Senior Compliance Manager, Health Futures, Microsoft Research	
Hugh Hemmings	Professor of Anesthesiology and Pharmacology, Weill Cornell Medicine	Editor-in-Chief, British Journal of Anaesthesia
Iyabo O. Muse	Associate Professor of Anesthesiology/ Division Chief of Regional Anesthesia and Acute Pain Medicine/ University of Virginia Health System	
Jenny Ferry	Consultant Anaesthetist, Aneurin Bevan UHB, UK	RA-UK board member, honoraria from Intelligent Ultrasound
Jennifer M. Weller	Professor and Head of Centre for Medical and Health Sciences Education, School of Medicine, University of Auckland, New Zealand<br>Hon Specialist Anaesthetist, Department of Anaesthesia and Perioperative Medicine, Auckland City Hospital, New	Editorial Board, British Journal of Anaesthesia<br>Executive Section Editor, Anesthesia and Analgesia

	Zealand<br>Director of Professional Affairs Education, Australian and New Zealand College of Anaesthetists	
Juan Pablo Miranda Pantoja	Sr Product Marketing Manager, Philips Ultrasound Inc	
Kariem El-Boghdadly	Consultant Anaesthetist, Guy's and St Thomas' NHS Foundation Trust; Honorary Reader, King's College London; Editor, *Anaesthesia*	Honoraria from GE Healthcare, PAION and Fisher and Paykel Healthcare.
Lopa Misra	Assistant Professor of Anesthesiology and Perioperative Medicine, Mayo Clinic, Phoenix, Arizona.<br>Associate Dean of Admissions, Mayo Clinic Alix School of Medicine, Phoenix, Arizona	
Marc Van de Velde	Consultant Anesthetist, Professor of Anesthesia, UZ Leuven and KU Leuven, Belgium	Lecture honoraria and consultancy honoraria from the following companies: CSL Behring, CSL Vifor, Viatris, Werfen, CAF-CDF, MSD.
Martin Benson	Head of Machine Learning, GE Healthcare	
Matthew Davies	Consultant Anaesthetist North West Anglia NHS Trust	Immediate Past President Association of Anaesthetists
Matthew Wiles	Consultant, Academic Department of Anaesthesia, Sheffield Teaching Hospitals NHS Foundation Trust, Sheffield; Honorary Fellow, Centre for Applied Health & Social Care Research (CARe), Sheffield Hallam University	Editor-in-Chief of *Anaesthesia*<br>Board member, Association of Anaesthetists
Melissa L. Byrne	Associate Professor of Anesthesiology, University of Virginia	
Mohamed Mostafa Mohamed	Consultant Anaesthetist ,<br>Lecturer of anaesthesia and pain medicine	

	Benha University Egypt<br>North Cumbria integrated care trust , Uk	
Nabil M Elkassabany	Professor of Anesthesiology, University of Virginia	
Nat Haslam	Consultant Anaesthetist, South Tyneside and Sunderland NHS Foundation Trust<br><br>President, Regional Anaesthesia-UK (RA-UK)	
Cailin Ng	CEO, HiCura Medical Pte. Ltd.	
Norman Kachel	VP Scientific Affairs & Innovation, B. Braun Melsungen AG	
Peter Merjavy	Consultant Anaesthetist, Craigavon Area University Teaching Hospital, Portadown, UK	ESRA-DRA Part II - Vice Chair, BBraun - honoraria
Rajnish K. Gupta	Professor of Anesthesiology, Vanderbilt University Medical Center	Board Member - American Society of Regional Anesthesia and Pain Medicine<br>Associate Editor - Regional Anesthesia and Pain Medicine
Rosemary M G Hogg	Consultant Anaesthetist, Belfast Health & Social Care Trust	Educational Honoraria GE Healthcare
Rupert M. Pearse	Professor of Intensive Care Medicine, Queen Mary University of London	
Samuel Gluck	Medical Administrator, NALHN, Adelaide, SA. Associate Senior Lecturer, University of Adelaide, SA	
Sandra L. Kopp	Professor of Anesthesiology and Perioperative Medicine Mayo Clinic Rochester MN	
Sebastian Layera	Assistant Professor, Department of Anesthesiology, Perioperative and Pain Medicine, University of Manitoba, MB, Canada	

Simeon West	Consultant Anaesthetist University College London Hospitals NHS Foundation Trust	
Simon Kos	Chief Medical Officer, Microsoft Australia New Zealand. Board member, Innowell.	
Stefan Mörl	Head of Pręֹ-Development	
Steve Margetts	Head of Data, GE Healthcare	
Suwimon Tangwiwat	Anesthesiologist, Department of Anesthesiology, Faculty of Medicine Siriraj Hospital, Mahidol University	
Tim Meek	(1) President, Association of Anaesthetists; (2) Consultant anaesthetist, James Cook University Hospital, Middlesbrough, UK	
Toby Ashken	Consultant Anaesthetist, University College London Hospital	
Tom. E. F. Abbott	Clinical Senior Lecturer in Anaesthesia & Perioperative Medicine, William Harvey Research Institute, Queen Mary University of London<br><br>Honorary Consultant, Department of Anaesthesia, The Royal London Hospital	In the last five years TA has been supported by an NIHR clinical lectureship and NIHR Development and Skills Enhancement Award;; has received research funding from Barts Charity, the Academy of Medical Sciences, The Royal College of Anaesthetists, and the British Journal of Anaesthesia; has received honoraria from MSD and Edwards Life Sciences; and is Social Media Editor of the British Journal of Anaesthesia.
Utku Kaya	Co-founder, SmartAlpha	
William Manson, MD, MBA	Medical Director of Perioperative Medicine<br>Associate Professor of Anesthesiology<br>UVA Health	Rivanna Medical
Veena Graff MD	Associate Professor of Anesthesiology & Critical Care, University of Pennsylvania Perelman School of Medicine	
