# Supplementary material for: Guidance for reporting artificial intelligence technology evaluations for ultrasound scanning in regional anaesthesia (GRAITE‐USRA): an international multidisciplinary consensus reporting framework
Source: Anaesthesia. 2025 Sep 18;80(12):1528–39. doi: 10.1111/anae.16733 (PMC12614416; doi:10.1111/anae.16733)
Supplement: Supplementary file 3 — Appendix S2. Glossary of terms. Appendix S3. Oxford University ethics waiver. Appendix S4. HRA Decision Tool. Appendix S5. GRAITE‐USRA longlist. Appendix S6. GRAITE‐USRA expanded checklist. [file ANAE-80-1528-s001.pdf]

Appendix S2 Glossary of terms

Term	Description
Artificial Intelligence (AI)	The simulation of human intelligence by machines, particularly computer systems. AI can perform tasks such as learning, reasoning, and problem-solving.
Classification	A type of machine learning task where the goal is to assign labels to inputs, such as categorising medical images as showing either a normal or abnormal finding.
De-identification	The process of removing or obscuring personal information from data to protect the privacy of individuals.
Delphi Consensus	A structured communication technique used in this study to reach an agreement among experts through rounds of surveys and feedback.
External Validation	Testing of the AI intervention on external data (collected in a different process to the training data, separated by time or space). Used to demonstrate generalisability of the AI intervention.
Feature Selection	The process of choosing which variables or data inputs to include in the AI model to improve its accuracy and efficiency.
Human Factors	How people interact with systems, including how they use, understand, and interpret outputs. This can include

	considerations such as user training and the ease of use of the AI system.
Internal Validation (Testing)	Testing of the AI model on holdout data (subset of data that is not used during the training of a model but is set aside to evaluate the model's performance) to satisfy developers that the AI model training is complete.
Machine Learning (ML)	A subset of AI that allows computers to learn from and make predictions or decisions based on data. ML models improve their performance over time as they are exposed to more data.
Missing Data	Information that is not available or was not collected during the study. Strategies to handle missing data include imputation (filling in missing values) or exclusion (removing incomplete data).
Model	In AI, a model is a mathematical representation of a system or process that has been trained on data to make predictions, classifications, or decisions based on new input data.
Model Outputs	The results or predictions generated by an AI model, such as whether a patient is at high or low risk for a certain condition.
Participant Flow Diagram	A chart that shows the progression of participants through the different stages of a study, from enrolment to analysis.

Performance Metrics	Measures used to evaluate the effectiveness of an AI model, such as accuracy, sensitivity, specificity, and area under the curve (AUC).
Primary Outcome	The main outcome measure that a study is designed to evaluate. This should be important to patients, clinicians and/or health organisations.
Reference Standard (Ground Truth)	The actual, real-world data or outcomes against which an AI model's predictions are compared to determine its accuracy.
Regional Anaesthesia	A type of anaesthesia that numbs a specific area of the body to prevent or reduce pain. It works by injection of local anaesthetic medication near specific nerves or groups of nerves.
Secondary Outcome	Additional outcome measures collected as part of a study but not the main focus.
Target Population	The specific group of people that the study or AI intervention is intended to impact, such as patients, clinicians, or other healthcare providers.
Training a Model	The process of teaching an AI model to recognise patterns or make decisions by exposing it to data and adjusting its internal parameters to minimise errors and improve accuracy.

Ultrasound Scanning	A medical technique that uses high-frequency sound waves to create images of structures within the body. In regional anaesthesia, ultrasound scanning is often used to guide needle placement and monitor the deposition of local anaesthetic medication.

Joint Research Office
enabling successful research through coll

UNIVERSITY OF OXFORD

NHS
Oxford University Hospitals
NHS Foundation Trust

Appendix S3 Oxford University ethics waver

Mr James Bowness
Department of Clinical Neurosciences
University of Oxford
by email

24 June 2024

To whom it may concern

GRAITE-USRA Guidelines for Reporting Artificial Intelligence Technology Evaluations for Ultrasound Scanning in Regional Anaesthesia (GRAITE-USRA)

This letter is to confirm that representatives of the Joint Research Office study classification group have reviewed the work referenced above.

It was determined that the activity proposed – Delphi survey of experts to establish consensus – is best understood as public engagement preparatory to service development or research. It is not subject to the Department of Health's *UK Policy Framework for Health and Social Care Research* (2017). It requires neither sponsorship nor research ethics review.

This opinion can be reviewed by reference to the HRA's algorithm, available at http://www.hra-decisiontools.org.uk/research/ and attendant leaflet, *Defining Research,* or by reference to The Health Care Quality Improvement Partnership (HQIP)'s *Guide for Clinical Audit, Research and Service review.*

Should you require further information, please do not hesitate to contact me.

Sincerely,

DocuSigned by:

Karen Melham

BA168DF4624B463...

Dr Karen Melham
Sponsorship and Ethics Lead

Research Services | Research Governance, Ethics & Assurance Team
University of Oxford, Boundary Brook House, Churchill Drive, Headington, Oxford OX3 7GB
T: 01865 616483 E: karen.melham@admin.ox.ac.uk

researchsupport.admin.ox.ac.uk

Go straight to content.

Medical Research Council

NHS Health Research Authority

Do I need NHS REC review?

To print your result with title and IRAS Project ID please enter your details below:

Title of your research:

```
Guidance for Reporting Artificial Intelligence Technology
Evaluations in Ultrasound Scanning for Regional Anaesthesia
(GRAITE-USRA)
```

IRAS Project ID (if available):

Your answers to the following questions indicate that **you do not need NHS REC review for sites in England.**

This tool only considers whether NHS REC review is required, it does not consider whether other approvals are needed. You should check what other approvals are required for your research.

You have answered **'YES'** to: Is your study research?

You answered **'NO'** to all of these questions:

Question Set 1

- Is your study a clinical trial of an investigational medicinal product?
- Is your study one or more of the following: A non-CE marked medical device, or a device which has been modified or is being used outside of its CE mark intended purpose, and the study is conducted by or with the support of the manufacturer or another commercial company (including university spin-out company) to provide data for CE marking purposes?
- Does your study involve exposure to any ionising radiation?
- Does your study involve the processing of disclosable protected information on the Register of the Human Fertilisation and Embryology Authority by researchers, without consent?

Question Set 2

- Will your study involve potential research participants identified in the context of, or in connection with, their past or present use of services (NHS and adult social care), including participants recruited through these services as healthy controls?
- Will your research involve prospective collection of tissue (i.e. any material consisting of or including human cells)

from any past or present users of these services (NHS and adult social care)?
- Will your research involve prospective collection of information from any past or present users of these services (NHS and adult social care)?
- Will your research involve the use of previously collected tissue and/or information from which individual past or present users of these services (NHS and adult social care), are likely to be identified by the researchers either directly from that tissue or information, or from its combination with other tissue or information likely to come into their possession?
- Will your research involve potential research participants identified because of their status as relatives or carers of past or present users of these services (NHS and adult social care)?

Question Set 3

- Will your research involve the storage of relevant material from the living or the deceased on premises in England, Wales or Northern Ireland without a storage licence from the Human Tissue Authority (HTA)?
- Will your research involve storage or use of relevant material from the living, collected on or after 1st September 2006, and the research is not within the terms of consent for research from the donors?
- Will your research involve the analysis of human DNA in cellular material (relevant material), collected on or after 1st September 2006, and this analysis is not within the terms of consent for research from the donor? And/or: Will your research involve the analysis of human DNA from materials that do not contain cells (for example: serum or processed bodily fluids such as plasma and semen) and this analysis is not within the terms of consent for research from the donor?

Question Set 4

- Will your research involve at any stage procedures (including use of identifiable tissue samples or personal information) involving adults who lack capacity to consent for themselves, including participants retained in study following the loss of capacity?
- Is your research health-related and involving offenders?
- Does your research involve xenotransplantation?
- Is your research a social care project funded by the Department of Health and Social Care (England)?
- Will the research involve processing confidential information of patients or service users outside of the care team without consent? And/ or: Does your research have Section 251 Support or will you be making an application to the Confidentiality Advisory Committee (CAG) for Section 251 Support?

If your research extends beyond **England** find out if you need NHS REC review by selecting the 'OTHER UK COUNTRIES' button below.

OTHER UK COUNTRIES

If, after visiting all relevant UK countries, this decision tool suggests that you do not require NHS REC review follow this link for final confirmation and further information.

Print This Page

NOTE: If using Internet Explorer please use browser print function.

Appendix S5 GRAITE-USRA initial longlist

SECTION 1 – TITLE

State the study design.

State that the intervention involves AI / machine learning.

SECTION 2 – ABSTRACT

Summary of relevant background, aims, methods, study type, main results, and conclusions.

SECTION 3 - INTRODUCTION

Describe the current practice, standard of care, or approach that represents the state of the art.

Describe pre-existing evidence for other AI interventions that fulfil the same purpose.

Provide rationale for the study.

State the study objectives or hypotheses.

SECTION 4 - METHODS

Report details of ethical approval, informed participant consent and compliance with regulatory requirements (e.g., trial registration).

Describe overall study design (e.g., type of study, prospective/retrospective).

Specify the evaluation stage (e.g. internal validation/testing or external validation) and provide details of the evaluation process.

Describe the study setting where the AI intervention was evaluated, including number and location of centres.

Describe any human factors considerations in study design (e.g., providing training for users, prior experience with AI intervention)

Describe eligibility criteria for participants (scan operators and scanned subjects).

Provide background information to give context about the AI intervention used, including type of AI, model outputs (e.g. classification, prediction) and version.

Describe the intended target population (e.g., clinicians as users of the intervention, patients as recipients of the intervention).

Define the metrics used to evaluate the AI model performance and explain why these specific metrics were chosen.

Describe the protocol for acquiring the ultrasound data, including details of the ultrasound machine manufacturer and transducer(s) used.

Define all primary and secondary outcome measures, including how and when they were assessed.

Describe the strategy to select features used for evaluation (e.g., block region, anatomical structures).

Define the reference standard (ground truth) used to evaluate the AI intervention, including the number and expertise of the sources or reviewers involved.

Describe methods to ensure data security and confidentiality (e.g., de-identification methods, data storage procedures).

Describe the methods for statistical analyses used to evaluate both primary and secondary outcomes, and explain the rationale for test selection.

Describe how sample size was determined (provide details of any sample size calculations, including clinical and statistical assumptions).

Provide information regarding patient and public involvement in the design, execution, reporting, interpretation, or sharing of the study results (or indicate if there was no involvement).

SECTION 5 - RESULTS

Define dates of data collection.

Consider including a results flow diagram demonstrating participant progress through the study phases (e.g., enrolment, allocation to intervention, follow-up, analysis), if appropriate.

Report data in a manner consistent with primary and secondary outcomes (including results of pre-specified statistical analyses).

Report ultrasound operator background information (e.g., level of training in regional anaesthesia).

Report patient/scan subject demographic information (including age, sex/gender, BMI, ethnicity).

Report missing data (e.g., lost data, non-adherence to protocol).

SECTION 6 - DISCUSSION

Describe the AI intervention's effect on its target population (e.g. patients, clinicians and other potential stakeholders).

Provide a general and balanced interpretation of results with reference to study objectives.

Place findings in context of previous studies/current landscape (including benchmarking against other available data if appropriate).

List the strengths/limitations of the AI intervention and potential facilitators/barriers to implementation.

Discuss implications and potential for future work.

Discuss any strengths/limitations relating to the study.

Provide a conclusion of the study, summarising the main findings and their potential impact.

SECTION 7 – OPEN SCIENCE

Use community-defined standards (e.g., Delphi consensus recommendations on nomenclature and anatomical structures).

Declare any conflicts of interest and financial disclosures for all authors.

Specify the source of funding and the role of the funders for the current study.

State where the study protocol can be accessed, or specify no protocol was prepared.

Provide information on where the study data and the analytical code can be accessed.

Provide details of the availability of the AI intervention or code. If it is not accessible, provide reasons why.

Appendix S6 Expanded GRAITE-USRA checklist

Section/topic	Item number	Checklist item and explanation
**TITLE**		
Title	1	**State the study design, specify that the study involves ultrasound scanning in the context of regional anaesthesia, and indicate that the intervention incorporates AI or machine learning.**<br>- Explicitly mentioning these elements facilitates accurate indexing, aids study identification, and supports future systematic reviews.
**ABSTRACT**		
Abstract	2	**Summary of relevant background, aims, methods, study type, main results and conclusions.**<br>- Identify the study as an evaluation of AI applications in ultrasound imaging for regional anaesthesia.<br>- Briefly outline the healthcare context, describe the AI intervention, and state the rationale for its evaluation.<br>- Specify the objectives of the study.<br>- State the study design.<br>- Describe the data sources and study setting.<br>- Summarise measures or criteria used to evaluate the AI intervention.<br>- Report the total number of study participants or datasets included.<br>- Provide a concise summary of the main outcomes.<br>- Provide an overall interpretation of the principal findings and their implications.
**INTRODUCTION**		
Background	3	**Describe the current practice, standard of care, or approach that represents the state of the art, and provide rationale for the study.**<br>- Summarise the current standard practice, clinical approach, or methodology relevant to the proposed AI intervention, referencing key literature as needed.<br>- Outline the healthcare context, specifying the intended clinical role or application of the AI technology under evaluation.<br>- Outline the healthcare context clearly, specifying the intended clinical role or application of the AI technology under evaluation.<br>- Explain how this study intends to address or advance the current standard of care or scientific understanding.
Objectives	4	**State the study objectives or hypotheses.**<br>- Explicitly state the main objectives, research questions, or hypotheses of your study.
**METHODS**		
Ethical approval	5	**Provide details of ethical approval, informed participant consent (or assent) and compliance with regulatory requirements (e.g. trial registration, data security).**

		- State whether the study received approval from an institutional research board or ethics committee, including the name of the approving body and approval reference number, if available.<br>- Describe how participant consent was obtained or specify if the ethics committee waived the requirement for informed consent.<br>- If ethical approval or informed consent was not required, explicitly state this, including the rationale.<br>- Provide details of how the study complied with relevant ethical guidelines and regulatory standard.<br>- Describe methods to ensure data security and confidentiality (e.g. de-identification methods, data storage procedures).
Study design	6	**Describe the overall study design (e.g. randomised controlled trial, cohort study, prospective/retrospective).**<br>- State the overall study design, specifying whether it was a randomised controlled trial, cohort study, or other study design.<br>- Indicate whether the study was conducted prospectively, retrospectively, or involved both prospective and retrospective elements.<br>- Provide details on relevant aspects of the chosen study design, including randomisation methods, allocation concealment, or cohort characteristics, if applicable.<br>- Justify the selection of the study design, demonstrating how it aligns with the stated objectives or hypotheses.
AI intervention	7	**Provide background on the AI intervention, including AI techniques used (e.g. deep learning), model outputs (e.g. classification, prediction) and version.**<br>- Provide information on the AI intervention, clearly identifying the specific AI methodologies employed (e.g. machine learning, deep learning).<br>- Outline the specific clinical or scientific context in which the AI model is applied, including relevant healthcare practices or technologies currently considered standard.<br>- Describe the intended role or purpose of the AI intervention within the clinical workflow (e.g. prediction, diagnosis, classification, or clinical decision support).<br>- State the software frameworks, including names and version numbers, to enable reproducibility.
Evaluation stage	8	**Specify the evaluation stage (e.g. internal validation/testing or external validation) and provide details of the evaluation process.**<br>- Indicate the evaluation stage conducted in the study (e.g. testing/internal validation using a held-out dataset or external validation using data from a different institution or context).<br>- Describe the methods and procedures employed during evaluation, including data partitioning approaches, and justify the rationale for these choices.<br>- Explain whether there were differences between datasets used in the development and evaluation phases, describing whether and how these were addressed or accommodated.
Participants	9	**Describe the study setting, including number and location of centres.**<br>- Describe the study setting, specifying whether it was conducted in a primary, secondary, or tertiary healthcare setting, research institution, or other relevant environment.

		- Report the number of centres involved and their geographical locations.<br>- If applicable, outline any differences in data collection or clinical practice across centres and how these were addressed in the study.<br>- Provide context on how the study setting relates to the intended application of the AI intervention and its potential generalisability.
	10	**Describe eligibility criteria separately for scan operators and scanned participants.**<br>- Define the eligibility criteria for scan operators, including relevant qualifications, training, experience required to perform ultrasound scanning, and familiarity with AI-assisted tools.<br>- Specify the inclusion and exclusion criteria for scanned participants, detailing relevant demographic, clinical, or procedural factors.<br>- If applicable, describe any restrictions on participant selection, such as age range, comorbidities, or prior exposure to ultrasound-guided procedures.<br>- Explain the rationale for the chosen eligibility criteria and how they align with the study objectives and intended application of the AI intervention.
Human factors	11	**Describe any human factors considerations in the study design (e.g. providing training for scan operators, prior experience with AI intervention)**<br>- Outline any training provided to scan operators, including its content, duration, and whether it was specific to the AI intervention.<br>- Specify the level of prior experience required for scan operators, including familiarity with ultrasound scanning and AI-assisted tools.<br>- Describe any measures taken to standardise operator performance, such as calibration sessions, protocols, or supervision.<br>- If applicable, detail how user interactions with the AI intervention were assessed, including usability testing or feedback collection.<br>- Explain how human factors were considered in study design to minimise variability and enhance the reliability of findings.
Target population	12	**Describe the intended target population (e.g. users and recipients of the AI intervention).**<br>- Define the intended target population, specifying both the users (e.g. clinicians, scan operators) and recipients (e.g. patients) of the AI intervention.<br>- Describe key demographic, clinical, or professional characteristics relevant to the AI intervention's application.<br>- If applicable, outline any subgroups within the target population that may experience different outcomes or require tailored considerations.<br>- Explain how the selected population aligns with the study objectives and the intended real-world implementation of the AI system.
Data	13	**Describe the protocol for acquiring the ultrasound data, including details of the ultrasound machine manufacturer(s) and transducer(s) used.**

		- Specify the ultrasound machine manufacturer(s), model(s), and software version(s) used in the study.<br>- Describe the type of transducer(s) employed, including frequency range and probe characteristics.<br>- Outline the protocol for ultrasound data acquisition, including standardised settings, imaging modes, and any preprocessing steps.<br>- If applicable, detail any operator-specific procedures or adjustments made during scanning.<br>- Explain how consistency in data acquisition was ensured across different operators, sites, or machines.
	14	**Describe or justify the strategy to select features used for evaluation (e.g. block region, anatomical structures).**<br>- Specify the features selected for evaluation, such as anatomical structures, block regions of interest, or ultrasound image characteristics.<br>- Justify the selection strategy, explaining whether features were chosen based on clinical relevance, prior research or other factors.<br>- Explain how consistency in feature selection was maintained across different scans, operators, or patient/participant groups.
Outcome	15	**Define all primary and secondary outcome measures, including how and when they were assessed.**<br>- Specify the primary outcome measure(s) and describe their clinical or technical relevance to the study objectives.<br>- Define any secondary outcome measures, explaining their role in supporting the primary analysis or providing additional insights.<br>- Describe how each outcome was assessed, including the methods, tools, or scoring systems used for evaluation.<br>- Indicate the time points at which outcomes were measured, ensuring alignment with the study design and intended application of the AI intervention.<br>- If applicable, explain any criteria for handling multiple outcome assessments, such as averaging repeated measures or prioritising specific time points.
Reference standard	16	**Define and justify the reference standard (ground truth) used to evaluate the AI intervention (e.g. the number and expertise of the sources or reviewers involved).**<br>- Specify the reference standard used to evaluate the AI intervention, detailing whether it was based on expert annotations, clinical outcomes, or other established criteria.<br>- Justify the choice of the reference standard, explaining its relevance and reliability in the context of ultrasound-guided regional anaesthesia.<br>- Provide details on the number, qualifications, and expertise of reviewers or annotators involved in establishing the reference standard.<br>- Describe any measures taken to ensure consistency and reduce bias, such as consensus review, adjudication processes, or interrater reliability assessments.<br>- If applicable, explain how discrepancies between reviewers were handled and whether any validation or benchmarking was performed against an independent standard.

Analysis	17	**Specify all measures used to evaluate the AI model performance and explain why these specific measures were chosen.**<br>- List all performance measures used to evaluate the AI model (e.g. area under the receiver operating characteristic curve (AUC-ROC), calibration metrics, sensitivity, specificity) as appropriate.<br>- Justify the selection of these measures based on the study objectives, clinical relevance, and the type of AI model being assessed.<br>- Explain how each measure provides insight into different aspects of model performance, such as scan accuracy, generalisability, or clinical applicability.<br>- If multiple metrics were used, describe how they complement each other to provide a comprehensive evaluation of the model.
	18	**Describe the methods for statistical analyses used to evaluate both primary and secondary outcomes.**<br>- Specify the statistical methods used to analyse both primary and secondary outcomes, including any tests, models, or comparisons applied.<br>- Justify the choice of statistical techniques, ensuring alignment with the study objectives and the nature of the data.<br>- Describe any adjustments made for potential confounders, or missing data to minimise bias.<br>- Report confidence intervals or other measures of statistical uncertainty.<br>- If applicable, explain how the statistical analysis accounted for variations across different subgroups or clinical settings.
Sample size	19	**Describe how the sample size was determined, including details of any calculations and the clinical and statistical assumptions used.**<br>- Explain the rationale for the chosen sample size, detailing whether it was based on a formal calculation, feasibility constraints, or prior studies.<br>- If a sample size calculation was performed, specify the statistical method used, including all estimates used in the calculation and any other key assumptions.<br>- Justify the clinical relevance of the selected sample size, ensuring it is sufficient to detect meaningful differences or achieve robust model evaluation.<br>- Describe any strategies used to account for potential biases, variability, or missing data in determining the sample size.<br>- If no formal calculation was conducted, provide a justification for the adequacy of the sample size in addressing the study objectives.
Adverse events	20	**Report how any adverse events were defined.**<br>- Define what constitutes an adverse event within the context of the study, specifying clinical, procedural, or technical criteria used for classification.<br>- Describe the severity grading system, if applicable, and indicate whether events were classified as minor, moderate, or serious.

		- Specify whether adverse events were predefined based on established guidelines or identified during the study period.<br>- If relevant, outline how adverse events were recorded, monitored, and reviewed to ensure consistency and accuracy in reporting.
Stakeholder involvement	21	**Provide details on any patient, public or stakeholder involvement in the design, execution, reporting, interpretation or sharing of the study results.**<br>- Describe any involvement of patients, the public, or stakeholders in the study's design, execution, or decision-making, including contributions to research questions, outcome selection, or ethical considerations.<br>- Outline participation in result interpretation and reporting, such as providing feedback on AI model usability, contributing to data analysis discussions, or shaping study conclusions.<br>- Specify any role in disseminating findings, including co-authorship, public engagement, or contributions to guideline development.<br>- If no involvement occurred, provide a rationale and discuss potential implications for study relevance and applicability.
**RESULTS**		
Data	22	**Report dates of data collection.**<br>- Specify the start and end dates of data collection, including any relevant recruitment timelines.
Participants	23	**Describe the flow of participants through the study (e.g. enrolment, allocation to intervention, follow-up, analysis), using a diagram if helpful for clarity.**<br>- Outline the progression of participants through key study stages, such as enrolment, allocation to intervention, follow-up, and analysis.<br>- Specify the number of participants at each stage, detailing any exclusions and reasons for dropout or data loss.<br>- Indicate whether participants were analysed according to their initial allocation or if any reclassification occurred.<br>- If applicable, describe differences in participant flow across study arms or subgroups.<br>- Consider using a flow diagram to visually represent participant progression and enhance clarity.
	24	**Report the characteristics on the scan operators (e.g. level of training in regional anaesthesia) and scanned participants (e.g. age, sex, BMI, ethnicity).**<br>- Provide details on the scan operators, such as their level of training, clinical experience in regional anaesthesia, and any prior exposure to AI-assisted ultrasound.<br>- Describe the demographic and clinical characteristics of scanned participants, such as age, sex, BMI, ethnicity, and any relevant medical history.<br>- If applicable, report subgroup distributions to highlight variability in operator expertise or participant characteristics.

		- Explain how these characteristics may influence the study findings, including potential implications for AI model performance and generalisability.
Outcomes	25	**Report results in a manner consistent with primary and secondary outcomes (including results of pre-specified statistical analyses).**<br>- Present results in alignment with the predefined primary and secondary outcomes, ensuring consistency with the study objectives.<br>- Report confidence intervals and p-values from the pre-specified statistical analyses to provide a clear interpretation of findings.
Missing data	26	**Report missing data (e.g. lost data, non-adherence to protocol).**<br>- Specify the extent of missing data for key variables, including the number and proportion of missing values.<br>- Describe the reasons for missing data, such as participant withdrawal, technical issues, or protocol deviations.<br>- Explain how missing data were handled in the analysis, and if applicable, assess the potential impact of missing data on study findings and generalisability.
Adverse events	27	**Report frequency and severity of any adverse events.**<br>- Provide the number and proportion of participants who experienced adverse events, categorising them by type and frequency.<br>- Describe the severity of adverse events.<br>- Indicate whether any adverse events led to study discontinuation, medical intervention, or serious complications.<br>- If relevant, compare adverse event rates across study groups or subpopulations to identify any patterns or risk factors.
**DISCUSSION**		
Interpretation	28	**Describe the key findings related to the AI intervention in the target population (e.g. patients, clinicians and other potential stakeholders).**<br>- Summarise the main findings of the AI intervention, focusing on its performance, effectiveness, or impact within the target population.<br>- Interpret results in the context of patients, clinicians, and other relevant stakeholders, highlighting potential benefits and limitations.
	29	**Provide a general and balanced interpretation of results with reference to study objectives.**<br>- Interpret the study findings in relation to the original objectives, ensuring alignment with predefined hypotheses or research questions.<br>- Present a balanced discussion of the results, ensuring that interpretations remain within the scope of the AI intervention's evaluation to avoid overinterpretation.
	30	**Place findings in context of previous studies/current landscape (including benchmarking against other available data).**

		- Compare the study findings with existing literature, highlighting similarities, differences, and potential reasons for any discrepancies.<br>- Benchmark results against previously published data, established models, or clinical standards to contextualise performance.<br>- Discuss how the AI intervention aligns with or advances current knowledge and practice within the field.<br>- If applicable, address gaps in the literature that this study helps to fill.
Strengths and limitations	31	**Discuss the strengths and limitations of the study and the AI intervention, including potential facilitators and barriers to implementation.**<br>- Discuss the strengths of the *study* and acknowledge its limitations, such as potential biases, sample size constraints, missing data, or factors affecting reproducibility and generalisability.<br>- Highlight the strengths of the *AI intervention* and address its limitations, such as performance variability, dependence on data quality, or potential challenges in real-world application.<br>- Identify key facilitators for implementation and discuss potential barriers, such as technical constraints, regulatory requirements, or the need for further validation across diverse clinical settings.
Future work	32	**Discuss implications and potential for future work.**<br>- Discuss potential implications for future research, such as whether additional evaluation is required to ensure robustness, reliability, and generalisability beyond the study cohort.<br>- Consider whether the model is ready for prospective clinical trials or if refinements are necessary before real-world implementation.
Conclusion	33	**Provide a conclusion of the study, summarising the main findings and their potential impact.**<br>- Summarise the main findings, highlighting the AI intervention's performance and its relevance to ultrasound-guided regional anaesthesia.<br>- Discuss the potential impact on clinical practice and future research, noting any limitations and the need for further evaluation or implementation.
**OPEN SCIENCE**		
Adherence to standards	34	**Adhere to community-defined standards (e.g. Delphi consensus recommendations on nomenclature and anatomical structures).**<br>- Use terminology, definitions, and anatomical references consistent with established community standards, such as the Delphi consensus recommendations for ultrasound-guided regional anaesthesia [1-4].<br>- Where deviations from these guidelines are necessary, provide justification and discuss potential implications for reproducibility and clinical application.
Conflicts of interest	35	**Declare any conflicts of interest and financial disclosures for all authors.**<br>- Disclose any financial relationships, industry affiliations, or potential conflicts of interest, or explicitly state if none exist.
Funding	36	**Specify the source of funding and the role of the funders for the current study.**

		- Report all funding sources and specify the role of funders in study design, data collection, analysis, interpretation, or manuscript preparation, or state if they had no involvement.
Protocol	37	**State where the study protocol can be accessed, or specify no protocol was prepared.**<br>- Specify availability of the study protocol, such as in a publication, supplementary material, or a public repository (e.g. Open Science Framework), including a URL or DOI if applicable.<br>- If no study protocol was developed or made publicly accessible, state this clearly and provide the reason.
	38	**Report any substantial amendments to the study protocol made after study commencement.**<br>- Summarise any significant deviations from the original protocol and explain the rationale for these changes.
Data	39	**Provide details of the availability of the data collected during the study. If they are not accessible, provide reasons why.**<br>- State whether the study data are available and specify where they can be accessed, such as a public repository, institutional database, or supplementary material, including a URL or DOI if applicable.<br>- If the data are not accessible, provide a rationale (e.g. legal constraints).
Code	40	**Provide details of the availability of the AI intervention and analytical code. If it is not accessible, provide reasons why (e.g. intellectual property protection).**<br>- Specify whether the AI intervention and analytical code are available, and provide details on where they can be accessed, such as a public repository (e.g. GitHub), institutional database, or supplementary material, including a URL or DOI if applicable.<br>- If the AI intervention or code is not accessible, explain the reasons (e.g. intellectual property protection, commercial restrictions, or data security concerns).

References:

1. Bowness JS, Pawa A, Turbitt L, et al. International consensus on anatomical structures to identify on ultrasound for the performance of basic blocks in ultrasound-guided regional anesthesia. *Reg Anesth Pain Med* 2022; **47:** 106-12. 10.1136/rapm-2021-103004
2. Ashken T, Bowness J, Macfarlane AJR, et al. Recommendations for anatomical structures to identify on ultrasound for the performance of intermediate and advanced blocks in ultrasound-guided regional anesthesia. *Reg Anesth Pain Med* 2022; **47:** 762-72. 10.1136/rapm-2022-103738
3. El-Boghdadly K, Albrecht E, Wolmarans M, et al. Standardizing nomenclature in regional anesthesia: An asra-esra delphi consensus study of upper and lower limb nerve blocks. *Reg Anesth Pain Med* 2023. 10.1136/rapm-2023-104884
4. El-Boghdadly K, Wolmarans M, Stengel AD, et al. Standardizing nomenclature in regional anesthesia: An asra-esra delphi consensus study of abdominal wall, paraspinal, and chest wall blocks. *Reg Anesth Pain Med* 2021; **46:** 571-80. 10.1136/rapm-2020-102451
